# Comparative performance of open thorax epicardial linear cryoprobe ablation versus focal tip radiofrequency catheter ablation in ventricular scar

**DOI:** 10.1007/s10840-026-02412-9

**Published:** 2026-07-24

**Authors:** Justin Hayase, Rami Aladham, Min Zhang, Gregory A. Fishbein, Takanori Sato, Shumpei Mori, Kalyanam Shivkumar, Olujimi A. Ajijola, Jason S. Bradfield

**Affiliations:** 1 UCLA Cardiac Electrophysiology Division, Ronald Reagan UCLA Medical Center, Los Angeles, CA USA; 2 UCLA Department of Pathology, Ronald Reagan UCLA Medical Center, Los Angeles, CA USA

**Keywords:** Cryoablation, Catheter ablation, Myocardial scar, Border zone

## Abstract

**Background:**

Epicardial cryoablation is increasingly utilized in open surgical procedures for treatment of ventricular arrhythmias in patients with structural heart disease when percutaneous epicardial access cannot be obtained. There are limited data regarding the effects of cryoablation within ventricular scar tissue, particularly in comparison to the performance of radiofrequency (RF) catheter ablation. This study aimed to investigate the comparative performance of ablation delivered epicardially in an open chest animal model with a linear cryoprobe versus a radiofrequency catheter.

**Methods:**

An infarct model was created in five Yorkshire swine using microsphere injection into the left anterior descending artery. After 6–8 weeks, animals underwent median sternotomy with targeted epicardial RF ablation lesions, and linear cryoablation lesions were delivered to myocardial infarct scar regions. Specimens were pressure perfused with 10% formalin, and histological analysis was performed.

**Results:**

Out of 25 epicardial RF lesions, 13 were visible and able to be analyzed. All 15 epicardial cryoablation lesions were visible. All 15 cryoablation lesions had transmural effect within scar where tissue thickness was ≤6 mm, whereas only 62% of evaluable RF lesions were transmural in scar (*p* = 0.01). Average cryoablation lesion depth was 4.2 ± 1.1 mm in scar and 5.6 ± 1.4 mm in border zone.

**Conclusion:**

Linear epicardial cryoablation is a useful tool in open surgical procedures to treat ventricular arrhythmias in structural heart disease. Cryoablation can achieve transmurality in tissue with ≤ 6 mm thickness with more technically reliable effect compared to catheter RF in the open chest.

**Supplementary Information:**

The online version contains supplementary material available at https://doi.org/10.1007/s10840-026-02412-9.

## Introduction

Catheter ablation is an important treatment option for patients suffering from ventricular arrhythmias. Recent randomized data support the use of catheter ablation over antiarrhythmic medications, even as an initial strategy [1]. In some patients, access to the epicardial surface is necessary to adequately treat the arrhythmogenic substrate. In patients with prior cardiac surgery or adhesions, percutaneous epicardial access is not possible, and instead, a surgical approach is required [2]. Radiofrequency (RF) ablation is a mainstay for endocardial ablation; however, this technology has more limited data to support its use in hybrid surgical procedures with an open chest setting [3, 4]. RF catheter ablation tools are familiar to cardiac electrophysiologists and have been adapted for use in hybrid surgical procedures because they integrate readily with electroanatomic mapping systems. However, there are technical constraints which challenge the use of RF including impedance limitations and catheter instability. Furthermore, extensive studies have established the performance of RF ablation in normal tissue; however, its effects in scar or border zone substrates are less predictable [5]. 

Cryoablation is an increasingly used tool in hybrid surgical procedures with observational data supporting its efficacy as well as some potential benefits compared to RF ablation tools including shorter procedure times and fewer complications [6, 7]. Benefits of cryoablation via a linear probe include its larger footprint, tissue adherence with greater stability and independence from tissue impedance. Prior work has described the lesion characteristics of epicardial cryoablation in an animal model [8, 9]. However, open chest cryoablation performance in scar tissue is not well-defined, particularly as compared to RF. In this study, we aimed to investigate the effects of epicardial cryoablation compared to catheter-delivered RF in a porcine infarct model in an open thorax setting.

## Methods

Animal experiments were conducted per the University of California Institutional Animal Case and Use Committee and the National Institutes of Health Guide for Care and Use of Laboratory Animals. All study protocols were approved by the UCLA Chancellor’s Animal Research Committee.

### Myocardial infarction creation

Myocardial infarction was induced as previously described [10]. Briefly, Yorkshire swine (Premier BioSource) weighing 30–35 kg (*n* = 5) were sedated with midazolam (1 mg/kg, intramuscular) and ketamine (10 mg/kg, intramuscular). After intubation, general endotracheal anesthesia was maintained using inhaled isoflurane (0.8– 1.5%), and carprofen (4.4 mg/kg, intramuscular) and buprenorphine (0.02 mg/kg, intramuscular) were administered for analgesia. Next, an over-the-wire angioplasty balloon (Armada 18, Abbott, Chicago, IL) was advanced through a femoral arterial sheath over the guide wire to the mid-LAD. The balloon was inflated, and a 10-ml suspension containing sterile saline and 4 ml polystyrene microspheres (Polybead, 90 μm, Polysciences, Warrington, PA) was slowly injected over 3–5 min. Surface ECG lead ST segment elevation confirmed immediate myocardial injury. Animals were recovered and six to eight weeks elapsed to allow maturation of the myocardial infarction prior to conducting terminal studies.

### Epicardial ablation

**Fig. 1:**
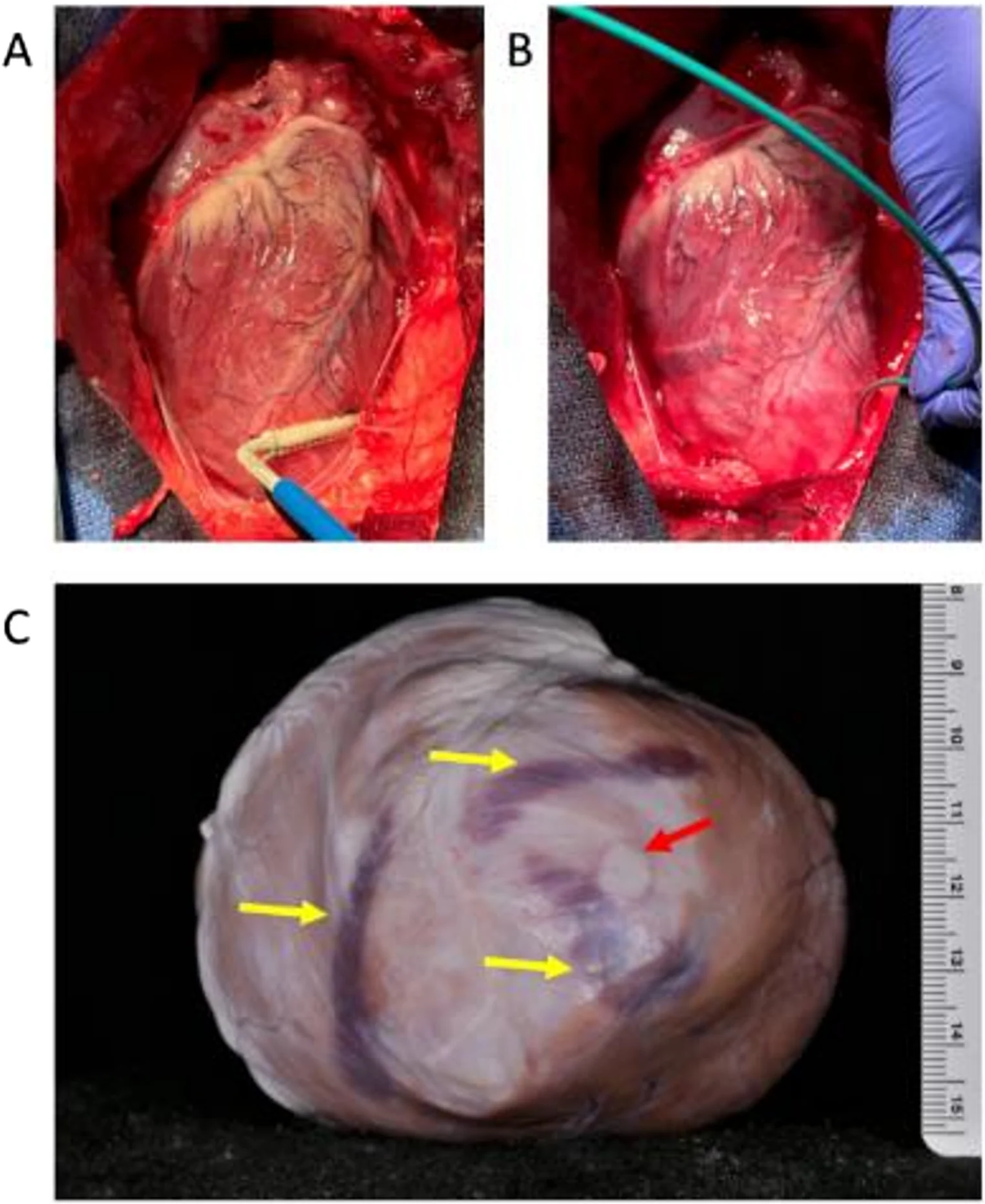
Gross specimen. (**A**) Cryoablation lesion delivery over the anterior left ventricle with the Cryoprobe (Atricure, Mason, OH), demonstrating the probe well-affixed to the epicardial surface during the freeze application. Cryoablation lesions were carefully delivered to span across dense scar and border zone tissue. (**B**) Radiofrequency ablation lesion delivery with a standard irrigated ablation catheter (Flexability, Abbott, Chicago, IL). (**C**) Gross specimen following fixation process. Cryoablation lesions are clearly seen as linear bands following fixation (yellow arrows). Radiofrequency lesions, when visible, with circular shape on the epicardial surface (red arrow)

Yorkshire pigs of either sex (50–60 kg) were used for terminal studies. The anesthetic regimen for terminal experimentation was sedation with Telazol (4–6 mg/kg intramuscular). Intubation was then performed with an isoflurane (1–2%) and fentanyl (100%g) intravenous bolus given once at the beginning of the preparation and intermittently during the study procedure. Median sternotomy was performed and the entire epicardial surface was exposed. A minimum of three cryoablation applications were performed for three minutes’ duration each using a CryoIce aluminum probe (Atricure, Mason, OH). The CryoIce probe utilizes N_2_O gas, and is designed with a malleable but rigid tip with smooth surface intended to maximize the probe-tissue interface and maintain stable contact throughout the duration of the lesion. The lesion duration was selected based upon prior animal study data showing diminishing return with regards to cryoablation depth beyond three minute lesions [8]. Care was taken to ensure lesions did not overlap and that each lesion visually spanned border zone and scar tissue (Fig. 1). Due to the larger footprint of the cryoablation probe, cryoablation lesions were delivered first, which then allowed for the focal RF lesions to be delivered without overlap. Subsequently, RF lesions were delivered epicardially using a standard irrigated ablation catheter (Flexability, Abbott, Chicago, IL). The chest cavity was filled with saline; however, due to technical constraints, it was not possible to fill the chest cavity sufficiently to completely submerge both the anterior and posterior myocardial surface. RF lesions were delivered at a power of 30–50 W for up to 30 seconds’ duration each. Longer duration RF was not performed due to significant challenges in catheter stability on the open epicardial surface (supplemental video). Care was taken to ensure RF lesions did not overlap with other RF or cryoablation lesions and were delivered in varying tissues of scar or border zone tissue, as space over the epicardial surface allowed. After completion of the experimental protocol, animals were euthanized with a lethal dose of potassium chloride.

### Specimen processing and histopathological analysis

**Fig. 2:**
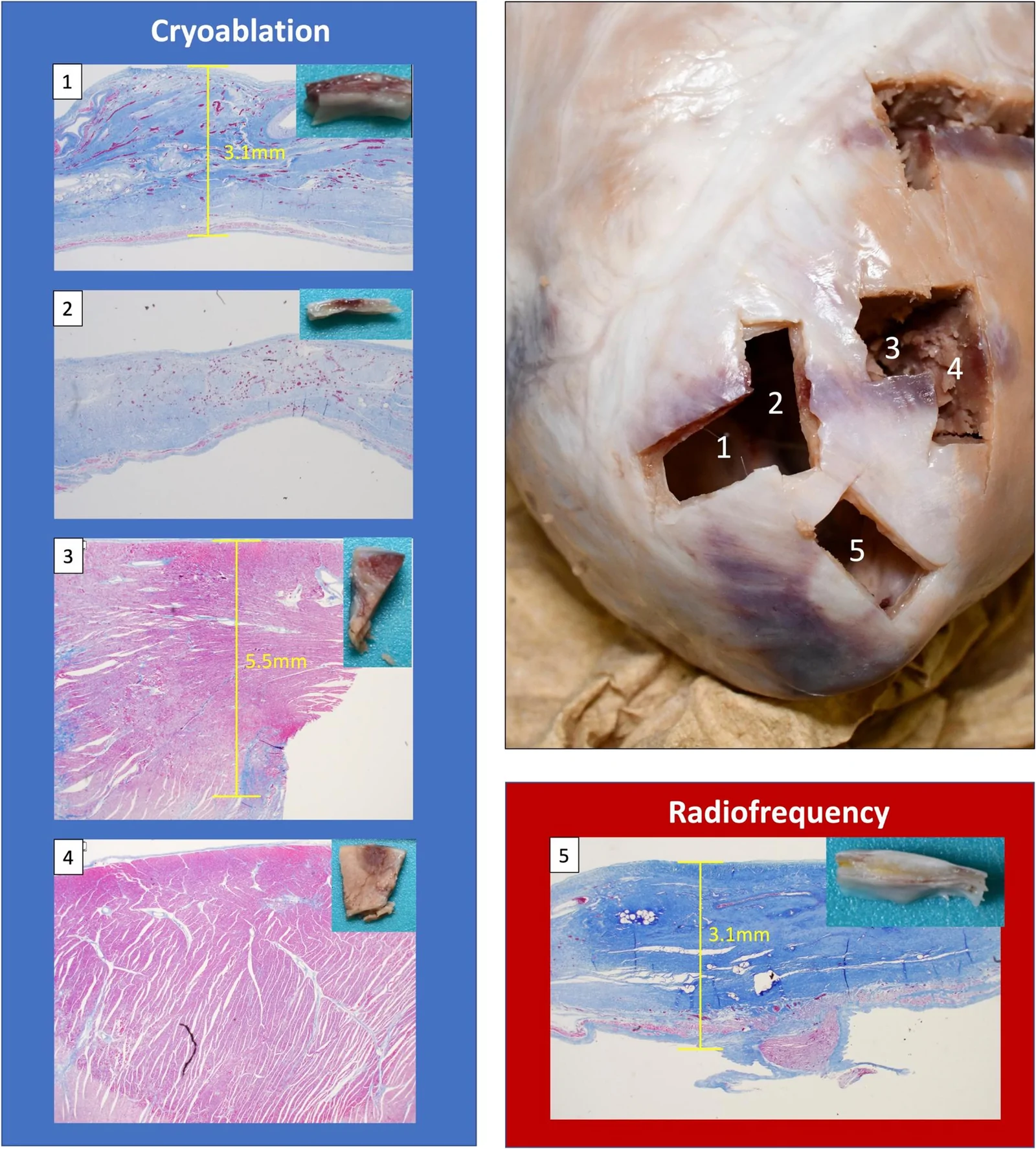
Left panel: Masson’s trichrome stained biopsy specimens of cryoablation lesions within scar (1 and 2) and border zone (3 and 4). Lesions were sectioned both perpendicular and parallel to the lesion orientation and maximal depth measurements were recorded. Top right panel: Gross specimen after fixation demonstrating corresponding biopsy sites. Lower right panel: Radiofrequency lesion Masson’s trichrome stain

The hearts were then extracted and pressure-perfused and fixated using flow pumps [11] using 10% formalin for 6-hr with careful modulation of pressure to achieve the same in-vivo end-diastolic dimensions. Following pressure-perfusion and fixation, the whole hearts were submerged in the 10% formalin for additional fixation for 5 days before wash and storage in the 0.01 mol/L phosphate buffered saline/0.02% sodium azide at 4 °C. Biopsies targeting the cryoablation and RF ablation lesions were performed, with particular attention to ensure sampling of border zone and scar tissue (Fig. 2). Formalin-fixed paraffin-embedded sections were stained with hematoxylin & eosin (H&E) and Masson’s trichrome. Measurements were made by two experienced cardiac pathologists (MZ, GF) utilizing Olympus cellSens Standard software.

## Results

There was a total of 15 cryoablation and 25 RF ablation lesions across five animals. Each animal accommodated three cryoablation lesions to the epicardial surface spanning scar and border zone – one to the anterior LV, one to the inferoapical LV, and one to the RV surface (Fig. 1C). Average cryoablation temperature was − 67.3 °C ± 1.1 °C. All cryoablation lesions were carried out to completion for the full three minutes each, with no arrhythmias, hemodynamic intolerance or other adverse effects.

The RF lesions were delivered for an average of 23.5 ± 14.0 s over scar regions. There were 16 RF lesions that had to be truncated due to steam pops, arrhythmia induction, or loss of tissue contact. Of the total 25 RF lesions, 13 were visible after specimen processing. All visible lesions were seen on the anterior surface of either the LV or RV. None of the inferiorly delivered lesions (0/6, 0%) were visible on ex vivo specimen analysis compared to the majority (13/19, 68%) being visible on the anterior surface. There were three steam pops which occurred, all at 50 W power delivery (3/8 attempts, 37.5%). These occurred early in the study period and subsequent RF lesions were delivered at 30 W and no further steam pops were observed. Of the remaining lesions, nine induced ventricular fibrillation (VF), and six were of limited duration due to excessive catheter instability. Only nine RF lesions were carried out to completion, with seven having no adverse immediate impact (no VF induced or steam pop).

### Histological analysis

**Fig. 3:**
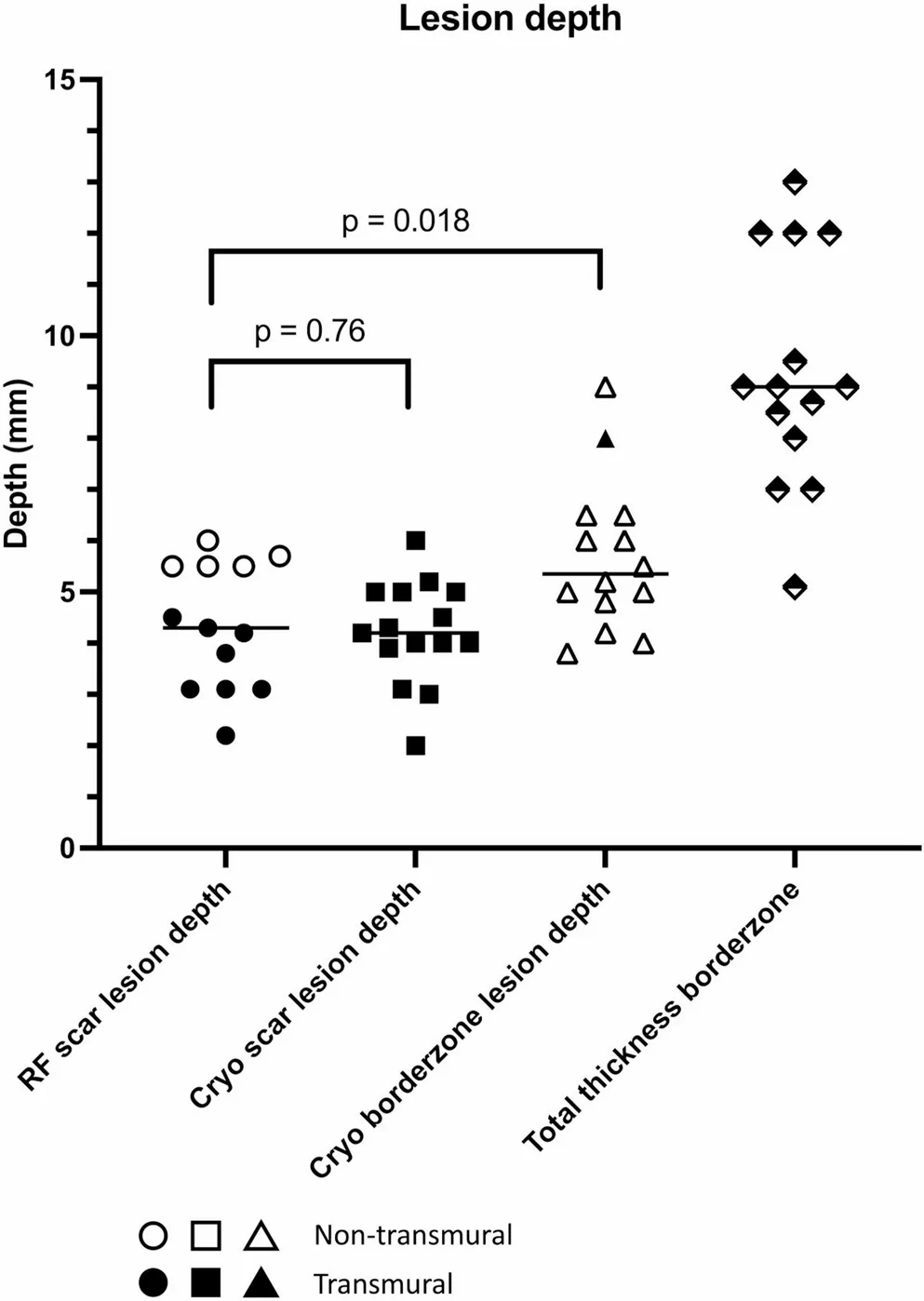
Ablation lesion depth within scar and borderzone tissue. There was no significant difference in overall depth of lesions in scar comparing radiofrequency and cryoablation; however, cryoablation more often achieved transmural effect in scar (100% versus 62%, *p* = 0.01)

**Fig. 4:**
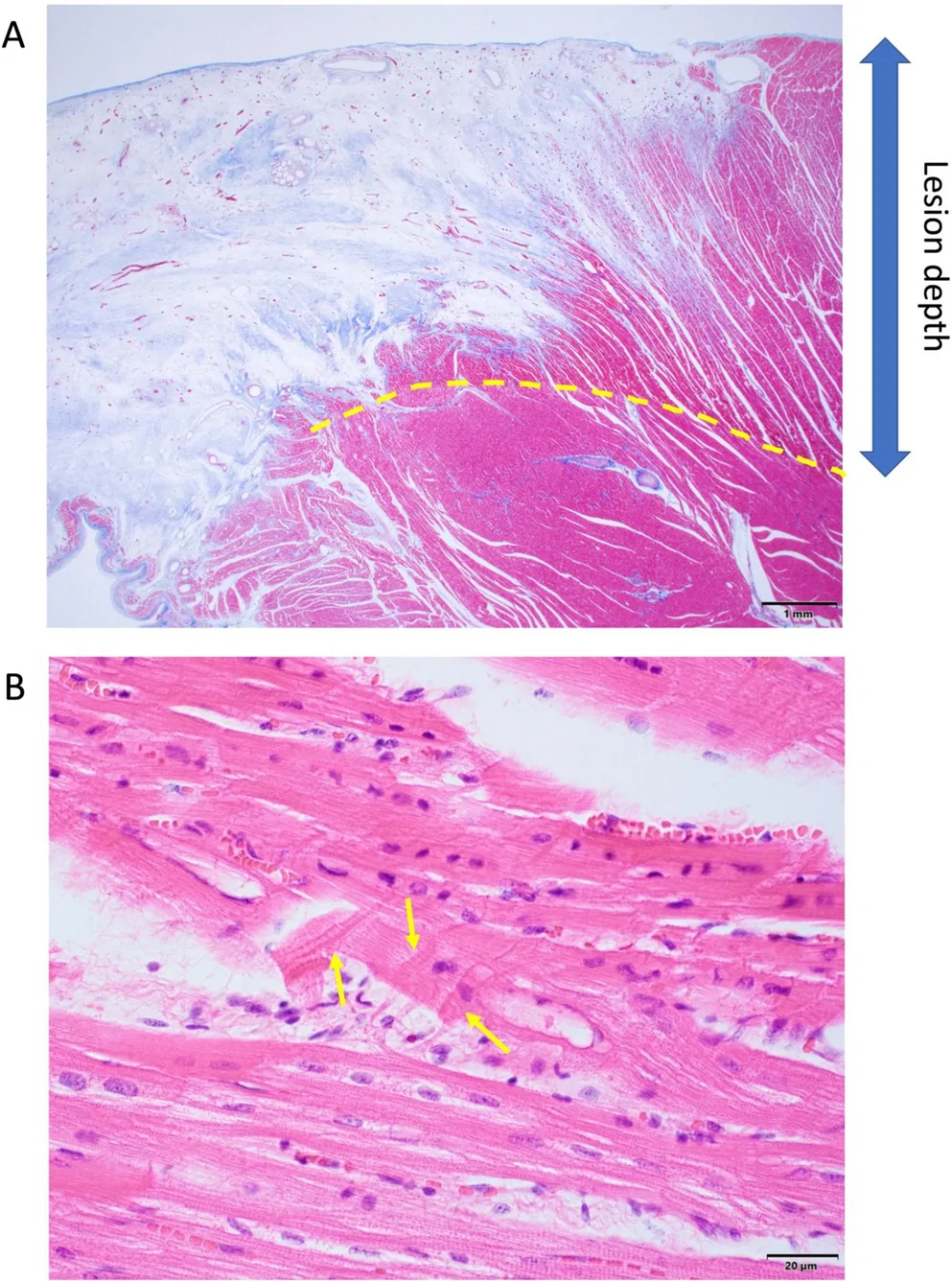
Cryoablation effect within scar borderzone. (**A**) Masson’s trichrome stain demonstrating an epicardial cryoablation lesion depth of 6 mm. Border of lesion is demarcated by yellow hatched line where markers of tissue necrosis such as contraction bands were clearly seen at higher power. (**B**) Hematoxylin and eosin stain at high power showing contraction bands (yellow arrows) indicative of myocardial necrosis within cryoablation lesion zone

All 15/15 (100%) cryoablation lesions were transmural in scar, compared to only 8/13 (62%) visible RF lesions which were transmural through scar (*p* = 0.01). The average depth of cryoablation lesions in scar was equal to the tissue thickness at that location (Fig. 3). Cryoablation lesions were transmural in regions where scar was 6 mm or less. Average cryoablation lesion depth was 4.2 ± 1.1 mm in scar, which was equivalent to the total tissue thickness in these regions. However, cryoablation lesions were only transmural in 1/16 lesions in border zone tissue regions of each cryoablation application where average tissue thickness was 9.3 ± 2.3 mm. Average cryoablation lesion depth was 5.6 ± 1.4 mm in border zone regions, and this depth was not limited by the presence of intervening myocardial fibrosis (Fig. 4). Of the 13 visible RF lesions, the average lesion depth was 4.3 ± 1.2 mm. Total tissue thickness depth was 5.3 ± 2.8 mm in these regions. Compared to the transmural cryoablation lesions in scar (4.2 ± 1.0mms), there was no significant difference in total tissue thickness (*p* = 0.2).

## Discussion

The findings of this study are:

1. Cryoablation with a linear probe is a more technically consistent ablation modality in the open chest compared to focal RF catheter ablation, with all lesions successfully carried out to completion.
2. Cryoablation delivered epicardially performs reliably within scar with predictable lesion effects.
3. Cryoablation delivered epicardially can achieve transmurality when tissue thickness is 6 mm or less.

RF catheter ablation remains a cornerstone for treatment of ventricular arrhythmias and is heavily utilized in percutaneous endocardial or epicardial mapping procedures. Ablation technology has made significant improvements with the addition of irrigated catheters, contact-force sensors, and monitoring of various ablation metrics including impedance changes to provide real time operator feedback to enhance lesion durability. However, these technologies were developed with the purpose of applying them via minimally invasive approaches. When attempting to employ these in an open surgical setting, there are numerous adjustments that need to be made in order to facilitate RF energy delivery, including modification of mapping system patch placement or filling the chest with saline in order to overcome impedance barriers [3]. Maintaining stable catheter contact is also very technically challenging. Cryoablation is a surgical tool that has been employed successfully in open chest ablation procedures [3, 7]. The cryoprobe can maintain stable contact once the probe has frozen into place, and the lesion delivery is not subject to impedance constraints that can limit RF delivery. The reliable completion of all cryoablation lesions and their consistent gross appearance are concordant with prior clinical experience demonstrating the practical advantages of surgical cryoablation over focal RF delivery in the open-chest setting [6]. 

Cryoablation lesions achieved transmural effect in regions of scar where tissue thickness was 6 mm or less. There are limited data regarding RF efficacy within scar, and even less data regarding cryoablation in scar or border zone. The tissue depth of lesions was somewhat attenuated in this study compared to the previous study in normal animals demonstrating cryoablation depth of up to 1.0 cm.^8^ There are multiple potential reasons for this. While transmural lesions were achievable with cryoablation, even in scar; the thermal distributive properties of scar are different than normal tissue. A greater water content in normal tissue may permit for improved cold temperature dispersion throughout the myocardial structure, compared to scar tissue. Furthermore, scarred myocardium is thinner than normal myocardium, which brings the endocardial blood pool relatively closer to the epicardially positioned cryoprobe, and this blood pool can serve as a heat sink which may lessen the effectiveness of the cryoablation. Lastly, the lesion depth measurements in this study were also limited as a function of the overall tissue depth, particularly in scar when transmurality was achieved. The tissue depth achieved is consistent with prior percutaneous studies of epicardial cryoablation, though this utilized different technology in a different anatomical setting [9]. 

The number of RF lesions which achieved transmurality was 62%, which is higher than that previously reported in other studies [5]. The number of visibly identifiable lesions was also higher compared to prior data; however, key differences include (1) this study was performed in an open chest model and (2) RF delivery was performed under direct visualization of the epicardial surface. Current density for RF delivery is higher in an open chest, which is evidenced by the higher percentage of visible RF lesions on the anterior surface of the heart, compared to the inferior surface. This also likely contributes to the high incidence of steam pops which occurred at high power. In addition, RF lesions were delivered under direct visualization on the epicardial surface of the heart which allowed for direct operator feedback as to efficacy of RF delivery and improved identification of RF lesion location for post-ablation analysis. Nonetheless, considerable technical challenges occurred with RF lesion delivery in this open chest study including contact instability and high incidence of steam pops with high power. These factors likely contributed to the comparatively superior performance of cryoablation in the surgical setting.

## Conclusions

Epicardial cryoablation is a useful tool for surgical procedures to treat ventricular arrhythmias. In an open chest setting, a linear cryoablation tool provides more predictable lesion formation compared to commonly used RF ablation catheters. Cryoablation achieved transmurality when tissue thickness is ≤ 6 mm, whereas RF did not achieve transmurality in 38% of evaluable lesions in this study.

## Limitations and future directions

Due to budgetary constraints, this was a relatively small animal study with surface area confinements on the epicardial surface limiting the number of ablation applications which could be studied. An additional limitation is that chronic animal infarcts matured for 6–8 weeks as is standard protocol at our institution. Although this time point is beyond the generally accepted period of infarct healing and scar maturation (> 1 month), during which the post-infarction substrate is considered relatively stable and reentrant ventricular tachycardia predominates, low-level remodeling of the fibrotic matrix persists indefinitely [12]. Therefore, the scar electro-architecture in this model may not fully recapitulate the substrate present in patients with longstanding chronic myocardial infarction. Other commercially available surgical RF devices have specific designs which allow for adherence to the epicardial surface and have flow directed irrigation toward the myocardium during RF application. It is likely that better RF delivery designs can overcome limitations of the open chest setting and achieve better results compared to the traditional catheter tools used in this study. Other specific RF surgical tools were unfortunately not available for use in this study; however, these catheter tools have been used clinically. In addition, pulsed field ablation (PFA) is a rapidly growing ablation modality, employed in percutaneous access ablation procedures. Whether PFA might offer other advantages over either RF or cryoablation in surgical access procedures should be the subject of future studies.

## Supplementary Information

Below is the link to the electronic supplementary material.

Supplementary Material 1 (MOV 3.68 MB)

## Data Availability

The data provided in this manuscript are the original results of a research project which was supported via an industry sponsored research grant and carried out at UCLA. Restrictions apply to the availability of these data.
